# The Association of Nonalcoholic Fatty Liver Disease With Neutrophil-to-Lymphocyte Ratio and Neutrophil-Percentage-to-Albumin Ratio

**DOI:** 10.7759/cureus.41197

**Published:** 2023-06-30

**Authors:** Dragoș Constantin Cucoranu, Marian Pop, Raluca Niculescu, Irina-Bianca Kosovski, Radu-Ovidiu Toganel, Razvan-Andrei Licu, Anca Bacârea

**Affiliations:** 1 Radiology, Mures County Emergency Hospital, Targu Mures, ROU; 2 Radiology, George Emil Palade University of Medicine, Pharmacy, Science and Technology of Targu Mures, Targu Mures, ROU; 3 Pathophysiology, George Emil Palade University of Medicine, Pharmacy, Science and Technology of Targu Mures, Targu Mures, ROU; 4 Doctoral School of Medicine and Pharmacy, George Emil Palade University of Medicine, Pharmacy, Science and Technology of Targu Mures, Targu Mures, ROU

**Keywords:** neutrophil-percentage-to-albumin ratio, neutrophil-to-lymphocyte ratio, non-alcoholic fatty liver disease, computed tomography, hepatic steatosis

## Abstract

Background and objective

Nonalcoholic fatty liver disease (NAFLD) is closely linked to metabolic syndrome, leading to consequences related to dyslipidemia, endothelial dysfunction, type 2 diabetes, and obesity. Due to a limited understanding of the factors contributing to the progression of NAFLD, predicting clinical outcomes in patients remains challenging. In light of this, this study aimed to evaluate the association between the occurrence of NAFLD and the neutrophil-percentage-to-albumin ratio (NPAR) as well as the neutrophil-to-lymphocyte ratio (NLR).

Methods

A total of 115 adult patients (mean age: 58 ± 12.5 years; 55.65% male) who underwent abdominal non-contrast-enhanced CT scans were included in the study. The analysis of CT scans was conducted to assess the attenuation values of liver parenchyma.

Results

There was a statistically significant difference in terms of gamma-glutamyl transpeptidase (GGT), triglyceride (TG), albumin, and NPAR between individuals with and without hepatic steatosis (GGT p<0.0001, TG p=0.0006, albumin p<0.0001, NPAR p=0.001). However, NLR values between the two groups did not show any statistical differences. NPAR (r=-0.27, p=0.0029) had a weak inverse correlation with liver attenuation value, which is expressed in Hounsfield units (HU).

Conclusions

Significant differences were observed in GGT, TG, albumin, and NPAR levels between individuals with and without hepatic steatosis. An inverse correlation between NPAR and liver attenuation values was also observed.

## Introduction

Individuals with nonalcoholic fatty liver disease (NAFLD) are at an increased risk of mortality from liver-related conditions, cardiovascular diseases, and cancer [1]. Since the factors causing NAFLD's progression have not yet been fully identified, predicting the clinical outcomes in this patient population is still difficult [2]. NAFLD is recognized as a manifestation of metabolic syndrome, whereby the clinical consequences are closely linked to conditions such as dyslipidemia, endothelial dysfunction, type 2 diabetes, and obesity [1,3,4]. Approximately 75-100% of obese persons develop NAFLD [5]. Even so, the mechanism behind the evolution from NAFLD to nonalcoholic steatohepatitis (NASH) is still unknown. Around 20% of people with NAFLD proceed to develop NASH, which is correlated with portal hypertension, cirrhosis, and hepatocellular carcinoma [6]. Early diagnosis and assessment of NAFLD and liver fibrosis are essential for monitoring disease progression and selecting the best treatment options for affected individuals [7]. The liver biopsy is the gold standard for the clinical diagnosis of hepatic steatosis and for determining the degree of liver fibrosis. However, due to its invasive nature, the procedure is inappropriate for evaluating a significant number of individuals who may be at risk or for monitoring hepatic steatosis patients who have undergone medical treatment. Furthermore, liver samples obtained from the biopsy are prone to sampling variability due to their small size [8]. Noninvasive imaging techniques have become more widely accepted as alternatives to liver biopsy [9]. CT enables the quantification of hepatic steatosis by assessing the liver attenuation value, which is expressed in Hounsfield units (HU). Since fat has a reduced attenuation value compared to soft tissue, hepatic parenchyma's attenuation value decreases as hepatic steatosis progresses [10].

Inflammatory pathways, specifically interleukin (IL)-1 type cytokines, play a critical role in the progression of hepatic steatosis and are linked to the prognosis of liver disease [7]. Even though cytokines are only rarely tested in routine practice, the total white blood cell count and its different subtypes might indicate the presence of an inflammatory condition [11]. The neutrophil-to-lymphocyte ratio (NLR) in particular has been suggested as an indicator of the risk for an inflammatory state in metabolic syndrome, cancer, and cardiovascular diseases [12-15]. Additionally, the neutrophil-percentage-to-albumin ratio (NPAR) is a useful biomarker that assesses systemic inflammation in a manner similar to the one described above by utilizing neutrophil percentage and albumin levels. Recent studies have shown a significant association between the presence of NAFLD and higher levels of NLR and NPAR. These findings suggest that these biomarker levels have the potential to be useful in predicting the progression of this disease [12,16,17]. Hence, the aim of this study is to assess the association between the occurrence of NAFLD and NPAR as well as NLR.

## Materials and methods

This cross-sectional, retrospective, single-center study was conducted in the Department of Radiology, Mures County Emergency Hospital, Targu Mures, Romania. The study included 115 adult patients who underwent abdominal non-contrast-enhanced CT at our facility between 2020 and 2021. Individuals under the age of 18 years, patients with known liver diseases other than steatosis, missing laboratory results, subpar CT scans, hemochromatosis, positive hepatitis B or C virus serology, and known alcoholism were all excluded. The study protocol followed all regulations stated in the Declaration of Helsinki and gained approval from the Ethics Committee of the Mures County Emergency Hospital (no: Ad.22273, dated September 9, 2022).

Demographic parameters (gender and age) along with laboratory findings [triglycerides (TG), total cholesterol, alanine transaminase (ALT), aspartate transaminase (AST), albumin, gamma-glutamyl transpeptidase (GGT), neutrophil count, and lymphocyte count] were documented from the institution's database and patient files. We collected the most up-to-date laboratory results obtained within a three-month timeframe prior to or following the CT scan. The NLR was calculated for each participant by dividing the total absolute neutrophil count by the total absolute lymphocyte count. The same blood test was used to compute NPAR by using the following formula: neutrophil percentage (%)/albumin (g/dL).

**Figure 1:**
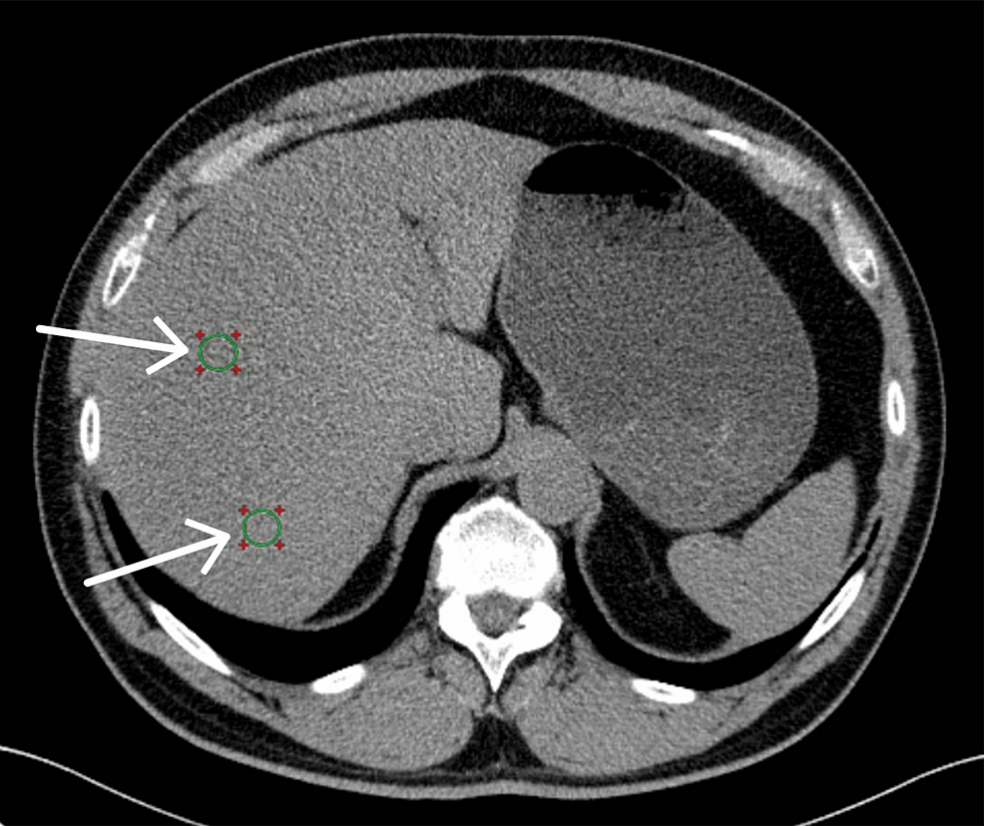
Example image of an axial non-contrast CT slice with the region of interest (ROI) placed within liver parenchyma (white arrows) CT: computed tomography

During a single breath-hold, an unenhanced abdomen CT examination was obtained on a 64-slice MDCT (Somatom Definition, Somatom Definition, Siemens Medical Solutions, Erlangen, Germany); 3 mm slices were obtained, using a tube voltage of 120 kV, and a tube current of 200 mA. Hepatic parenchyma hypoattenuation with an absolute density of under 48 HU, as suggested in previous studies in the literature [18,19], was adopted as the hepatic steatosis diagnostic criteria. Hepatic attenuation was measured, using Radiant software, by averaging the attenuation values for eight 1.5 cm^2^ regions of interest (ROI) positioned in distinct axial slices within hepatic segments V, VI, VII, and VIII (Figure 1) defined based on the Couinaud system. Utmost caution was taken when placing each ROI to prevent the inclusion of macroscopic hepatic vessels. The intraobserver variability was determined using two measurements by the same radiology trainee.

The statistical analysis involved calculating means, standard deviations (SD), medians, interquartile ranges (IQR), and counts and percentages, depending on the distribution of the parameters. Continuous variables were compared using Mann-Whitney U tests and unpaired t-tests. The χ^2^ test was employed to compare categorical variables. Spearman's correlation coefficients were used to examine the relationship between NLR, NPAR, and liver HU. The study employed established criteria for correlation strength, categorizing it as none (0.0-0.1), weak (0.1-0.3), moderate (0.3-0.5), or high (0.5-1.0). For the assessment of intraobserver variability in continuous variables, the intraclass correlation coefficient was used. Statistical analysis was conducted by using GraphPad Prism version 9.1 (GraphPad Software, San Diego, CA).

## Results

The study included a total of 115 participants. The mean age of the cohort was 58 ± 12.5 years and 55.65% were male; 73 individuals (63.47%), with liver HU of 42.9 ± 12.8 had hepatic steatosis. Measures of liver density showed excellent intraobserver reliability, which had an intraclass correlation value of 0.99. There was a statistically significant difference in terms of GGT (p<0.0001), TG (p=0.0006), albumin (p<0.0001), and NPAR (p=0.001) levels between individuals with and without hepatic steatosis. However, the NLR values between the two groups did not show any statistical differences. The characteristics of the study participants are shown in Table 1. Moreover, NPAR (r=-0.27, p=0.0029) had a weak inverse correlation with liver HU (Table 2).

**Table 1:** Characteristics of the study cohort *Statistically significant SD: standard deviation; IQR: interquartile range; AST: aspartate transaminase; ALT: alanine transaminase; GGT: gamma-glutamyl transpeptidase; TG: triglycerides; NLR: neutrophil-to-lymphocyte ratio; NPAR: neutrophil-percentage-to-albumin ratio

	Hepatic steatosis	No hepatic steatosis	P-value
	N=73	N=42	
Gender, n (%)			0.18
Male	44 (60)	20 (48)	
Female	29 (40)	22 (52)	
Age, mean ± SD	59.9 ± 11	55.1 ± 14	0.06
Laboratory parameters, median (IQR)			
ALT, U/L	26.3 (16.9-43.5)	21.4 (13.3-33.8)	0.8
AST, U/L	26 (18.5-35.7)	20.1 (15.9-30.2)	0.06
GGT, U/L	108 (43-183.5)	37 (19-68.5)	<0.0001*
TG, mg/dL	143.6 (102.8-181.1)	101.5 (84.6-141.9)	0.0006*
Total cholesterol, mg/dL	183.7 (150.1-216.7)	165.4 (138.3-197.4)	0.1
Albumin, g/dL	3.8 (3.1-4.2)	4.3 (4-4.6)	<0.0001*
NLR	2.8 (1.8-4.6)	2.6 (1.7-3.4)	0.3
NPAR	18.2 (14.2-21.4)	15.1 (12.3-17.4)	0.001*
Lymphocyte count, 10^3^/µL^3^	1.7 (1.2-2.4)	1.9 (1.5-2.4)	0.1
Neutrophil count, 10^3^/µL^3^	5.3 (3.8-7.2)	4.8 (3.5-7.8)	0.9
Neutrophil, %, mean ± SD	65.2 ± 11	64.4 ± 10	0.7

**Table 2:** Correlation between liver attenuation values and NLR and NPAR *Statistically significant HU: Hounsfield unit; NLR: neutrophil-to-lymphocyte ratio; NPAR: neutrophil-percentage-to-albumin ratio

	Liver attenuation values (HU)	
	r	P-value
NLR	-0.05	0.53
NPAR	-0.27	0.0029*

## Discussion

The objective of this retrospective cross-sectional study was to investigate the potential association between the presence of NAFLD and NPAR and NLR. We found an association between NPAR and the occurrence of NAFLD. Moreover, the NPAR levels were significantly inverse-correlated with the liver attenuation values.

Although several laboratory indicators of systemic inflammation have been studied as predictive biomarkers in NAFLD, their clinical use is frequently precluded by cost concerns and technical challenges [20]. Peripheral blood leukocyte measurements and counts, particularly of neutrophils as part of NLR, serve as a low-cost and often-used technique for determining whether inflammation is present. Increased NLR values in NAFLD patients are brought on by inflammation that develops as a result of systemic lipotoxicity resulting from lipid metabolites, insulin resistance, the release of proinflammatory cytokines including IL-6 and tumor necrosis factor (TNF)-α, as well as oxidative stress and adipokines [21]. Albumin, which constitutes over 50% of the total human serum content, is a moderately-sized protein that serves various roles, such as an anti-inflammatory agent, regulating osmotic pressure, and as an antioxidant. There are particular changes to albumin's function and structure that are linked to diseases like cirrhosis in addition to decreased albumin production [22]. In patients with septic shock [23], acute renal injury [24], and palliative pancreatic cancer [25], NPAR, which integrates albumin and NLR, is employed as a systemic inflammation predictor. Additionally, NPAR is linked to mortality among individuals with liver cirrhosis and atrial fibrillation [26,27]. We observed a similar significant association between NPAR and the presence of NAFLD in a recent study by Liu et al. They reported a statistically significant correlation (14.3 ± 0.1, p=0.013) between NPAR and NAFLD, and these results are in line with our findings [16].

The association between NLR and the presence of hepatic steatosis has been the subject of debate in several recent studies. Some studies have reported a statistically significant relationship [12,17,28], indicating that NLR values were higher in individuals with hepatic steatosis. However, contrasting findings have also been reported in certain other studies, where no significant association between NLR and hepatic steatosis was observed [29,30]. Zhou et al. [30] found that NLR was not significantly correlated with NAFLD. In contrast, a recent study focusing on assessing the diagnostic efficacy of the NLR as a marker for steatosis observed that NLR exhibited a significant positive correlation with the intensity of steatosis [21]. The study highlighted the high sensitivity and specificity of NLR as an assessment tool. However, in our study, we did not identify a notable association between NLR and the occurrence of steatosis. Consequently, additional investigation is warranted to delve deeper into this subject and gain a more complete understanding. Chronic inflammation plays an important role in the development of NAFLD, which may range from simple steatosis to NASH, advanced liver fibrosis, cirrhosis, and hepatocellular carcinoma [17]. The ability to noninvasively identify and track progressive liver disease is crucial since NAFLD is a significant contributor to the progression of hepatic injury.

Our study has certain limitations that should be taken into account. The study design was cross-sectional and retrospective, and it was conducted at a single center. Longitudinal studies with a larger, more diverse population would be beneficial to further explore the associations observed in our study. Additionally, the use of a single center for data collection introduces the potential for selection bias, limiting the generalizability of the findings to other settings. Long-term follow-up studies would also be beneficial in assessing the dynamic nature of NAFLD and its associated factors. By addressing these limitations, we can further advance our understanding of NAFLD and its implications for clinical practice and public health.

## Conclusions

Our study found a statistically significant association between NPAR and the presence of NAFLD as assessed by non-contrast-enhanced CT scans. Furthermore, NPAR had an inverse correlation with the liver HU values. However, we did not find any association between NLR and the occurrence of NAFLD in our study.
